# Serologic Evidence of Influenza A (H14) Virus Introduction into North America

**DOI:** 10.3201/eid2112.150413

**Published:** 2015-12

**Authors:** Neus Latorre-Margalef, Andrew M. Ramey, Alinde Fojtik, David E. Stallknecht

**Affiliations:** University of Georgia, Athens, Georgia, USA (N. Latorre-Margalef, A.M. Ramey, A. Fojtik, E. Stallknecht);; Lund University, Lund, Sweden (N. Latorre-Margalef);; US Geological Survey Alaska Science Center, Anchorage, Alaska, USA (A.M. Ramey)

**Keywords:** influenza, influenza A, influenza virus, viruses, host, ducks, wild birds, subtype, H14, distribution, hemagglutinin, serology, zoonoses, North America

**To the Editor:** Although a diverse population of influenza A viruses (IAVs) is maintained among ducks, geese, shorebirds, and gulls, not all of the 16 avian hemagglutinin (HA) subtypes are equally represented (*1*). The 14th HA subtype, commonly known as the H14 subtype, was historically limited to isolates from the former Soviet Union in the 1980s (*2*) and was not subsequently detected until 2010, when isolated in Wisconsin, USA from long-tailed ducks and a white-winged scoter (*3*–*5*). In the United States, the H14 subtype has since been isolated in California (*6*), Mississippi, and Texas (*7*); and has been reported in waterfowl in Guatemala (*7*). In this study, we examined whether there was serologic evidence of H14 spread among ducks in North America before (2006–2010) and after (2011–2014) the initial detection of the H14 subtype virus on this continent. 

This report was reviewed and approved by United States Geological Survey under the Fundamental Science Practices policy (http://www.usgs.gov/fsp/). Serum samples from blue-winged teal, American green-winged teal, and mallard ducks were screened by using blocking ELISA (FlockCheck AI MultiS-Screen antibody test kit; IDEXX Laboratories, Westbrook, ME, USA) to detect antibodies against the influenza virus nucleoprotein. Positive samples were tested by microneutralization assays as described (*7*) against viruses representing H14 and H3 subtypes. H3 is commonly detected in ducks found in North America (*8*) (online Technical Appendix Table 1, http://wwwnc.cdc.gov/EID/article/21/12/15-0413-Techapp1.pdf).

Antibodies against H3 were detected during 2006–2014 in Michigan, Minnesota, New Jersey, Texas, and Louisiana (Table); titers ranged from 20 to 320. Antibodies against H14 were detected in 1 duck in 2007 and in 24 ducks sampled in 2012 after August. H14 antibodies were detected in all years and most locations studied after 2012; antibody titers ranged from 20 to 160. Thus, antibody prevalence was consistent with the relative prevalence of H3 reported among ducks in North America (*1*,*4*,*8*) and the timing of initial detection of H14 viruses.

To address the possibility of cross-neutralizations between HA subtypes, we tested the 2007 H14-positive serum samples and 22 of the H14-positive serum samples from 2012–2014 against HA subtypes 1–12 (online Technical Appendix Table 1) by virus neutralization (online Technical Appendix Table 2). Among humans, broadly neutralizing antibodies within HA groups targeting conserved regions in the HA stalk have been described (*9*), and if present in samples from mallards, these could contribute to cross-neutralizations. The H14-positive serum samples from 2007 reacted to subtypes H3, H4, H7, and H11, and high titers were identified for H3 and H4, which are within the same clade. Samples from 17 of these birds tested antibody-positive for additional HA subtypes and 5 tested positive only to H14. An H14 virus was recovered by virus isolation from the same blue-winged teal population sampled in March 2013, from which serum samples were obtained (*7*); however, although H14 antibodies have been detected in Minnesota, an H14 virus has not yet been isolated in that state.

Our serologic results are temporally consistent with H14 isolation reports and suggest that H14 subtype viruses were not circulating among ducks in North America before initial virus isolation. However, there are potential challenges with serologic-based investigations. For example, the overall prevalence of H14 antibodies after the initial detection of H14 viruses (2011–2014) was low (3.5% of blocking ELISA positive samples), thus requiring a large sample size (n = 670) for H14 antibody detection. However, an even lower prevalence was observed by using virus isolation; we isolated only 1 H14 IAV during parallel sampling of these sites (n = 8,875) during 2011–2014.

Differences in pre- and post-H14 detection also varied between species, location, and season. Differences in H14 antibody prevalence were observed in all ducks sampled pre- and post- (0.3%–3.5%, p = 0.0103) H14 detection, but not in the mallard-only subset (0.3%–2.1%, p = 0.0963). A significant difference in seroprevalence also was detected between species (mallard [2%] vs teal [6%]) in the 2011–2014 samples (p = 0.0104). IAV show strong seasonal patterns in prevalence, and the observed differences in antibodies may be associated with the probability of IAV infection before sampling and the persistence of antibody responses in these species. Mallards (primarily hatch-year birds) were sampled at the beginning of fall migration (≈3–4 months of potential IAV exposure for hatch-year birds), whereas teal were sampled later, during spring migration (≈9–10 months of potential IAV exposure for birds hatched the previous spring or summer). It is apparent that the sampling approach used can affect results.

Interpretation of subtype-specific serologic data can be complex, especially in birds that are normally infected with several IAV subtypes during their lives. Nevertheless, this study demonstrates the value of a subtype-specific serologic approach to detect even relatively minor changes in subtype diversity and clearly shows that new viruses can establish in duck populations in North America. Serologic techniques also can be optimized to detect incursions of novel viruses such as the highly pathogenic Eurasian H5 viruses (*10*) among wild birds.

**Table:** H3 and H14 microneutralization assay data from ducks sampled during 2006–2014, North America

Year	Month of sampling	State	Species	No.	H3N8, no. (%)	H14N5, no. (%)
2006	Aug	Michigan	Mallard	29	6 (21)	0
	Aug/Sep	Minnesota	Mallard	39	3 (8)	0
2007	Aug/Sep	Minnesota	Mallard	46	8 (17)	1 (2)
2008	Aug/Sep	Minnesota	Mallard	44	8 (18)	0
2009	Aug/Sep	Minnesota	Mallard	29	10 (34)	0
	Aug	New Jersey	Domestic and wild mallard	36	1 (3)	0
2010	Aug/Sep	Minnesota	Mallard	29	6 (21)	0
	Aug	New Jersey	Domestic and wild mallard	20	5 (25)	0
2011	Aug/Sep	Minnesota	Mallard	124	37(30)	0
2012	Feb/Mar	Texas	Blue-winged teal	19	3 (16)	0
	Aug/Sep	Minnesota	Mallard	188	11 (6)	2 (1)
2013	Feb/Mar	Texas/Louisiana	Blue-winged teal	120	13 (11)	12 (10)
	Feb/Mar	Texas/Louisiana	American green-winged teal	91	5 (5)	2 (2)
	Aug/Sep	Minnesota	Mallard	65	8 (12)	7 (11)
2014	Feb/Mar	Texas	Blue-winged teal	22	1 (5)	1 (5)
	Sep	Minnesota	Mallard	41	4 (10)	0
Totals						
2006–2010	NA	NA	All ducks	272	47 (17)	1 (0.3)
	NA	NA	Mallards only	272	47 (17)	1 (0.3)
2011–2014	NA	NA	All ducks	670	82 (12)	24 (3.5)
	NA	NA	Mallards only	418	60 (14)	9 (2.1)
	NA	NA	Blue-winged teal and American green-winged teal	252	22 (9)	15 (6)

*NA, not applicable.

**Table:** 

**Technical Appendix.** Antigens used for microneutralization assays and subtype-specific virus neutralization data for H1–H12 from 23 birds positive for H14.
