# Supplementary material for: Serologic Evidence of Influenza A (H14) Virus Introduction into North America
Source: Emerg Infect Dis. 2015 Dec;21(12):2257–9. doi: 10.3201/eid2112.150413 (PMC4672416; doi:10.3201/eid2112.150413)
Supplement: Supplementary file 1 — Technical Appendix. Antigens used for microneutralization assays and subtype-specific virus neutralization data for H1–H12 from 23 birds positive for H14. [file 15-0413-Techapp-s1.pdf]

Article doi: http://dx.doi/10.3201/eid2112.150413

Serologic Evidence of H14 Influenza A Virus Introduction into North America

Technical Appendix

Technical Appendix Table 1. List of antigens used for microneutralization assays of samples from ducks, North America

A/mallard/MN/AI0-4823/2010 (H1N1)
A/mallard/MN/AI08-2755/2008 (H2N3)
A/mallard/MN/AI0-2593/2010 (H3N8)
A/mallard/MN/AI10-3208/2010 (H4N6)
A/mallard/MN/AI11-3933/2011 (H5N1)
A/mallard/MN/AI08-2721/2008 (H6N1)
A/mallard/MN/AI08-3770/2009 (H7N9)
A/mallard/MN/SG-01048/2008 (H8N4)
A/RUTU/DE/AI11-809/2011 (H9N2)
A/mallard/MN/SG-00999/2008 (H10N7)
A/mallard/MN/SG-00930/2008 (H11N9)
A/mallard/MN/SG-3285/2007 (H12N5)
A/blue-winged teal/TX/AI13-1028/2010 (H14N5)

Technical Appendix Table 2. Subtype-specific virus neutralization data for H1–H12 from 23 birds positive for H14, North America*

					Group 1								Group 2				
					H9 Clade			H1 Clade				H11 Clade	H7 Clade		H3 Clade		
Date		Sample ID	Age†	Species‡	H9N2	H8N4	H12N5	H5N1	H2N3	H1N1	H6N1	H11N9	H7N9	H10N7	H3N8	H4N6	H14N5
2007																	
	Sep 17	AI07-4825	UA	MALL	<20§	<20	<20	<20	<20	<20	<20	**160**	**80**	<20	**640**	**640**	**40**
2012																	
	Sep 17	AI12-2725	HY	MALL	<20	<20	**80**	<20	<20	<20	<20	<20	<20	<20	<20	<20	**40**
2013																	
	Mar 1	AI13-112	AHY	BWTE	<20	<20	**40**	<20	<20	<20	<20	**20**	<20	**40**	<20	<20	**40**
	Mar 1	AI13-114	AHY	BWTE	**20**	**320**	<20	**20**	<20	<20	<20	20	<20	<20	**40**	<20	**40**
	Mar 1	AI13-137	AHY	BWTE	<20	<20	<20	<20	<20	<20	<20	<20	<20	<20	<20	<20	**20**
	Mar 1	AI13-6	AHY	BWTE	<20	<20	<20	<20	<20	<20	<20	**20**	<20	<20	<20	<20	**20**
	Mar 5	AI13-393	HY	BWTE	**80**	<20	**640**	**640**	**640**	**640**	**640**	<20	<20	**160**	<20	**40**	**160**
	Mar 5	AI13-433	AHY	AGWT	<20	<20	<20	<20	<20	20	<20	<20	<20	<20	<20	<20	**20**
	Mar 6	AI13-511	AHY	BWTE	<20	<20	<20	**40**	<20	<20	**40**	<20	<20	<20	<20	<20	**20**
	Mar 6	AI13-528	AHY	BWTE	<20	<20	**320**	<20	<20	<20	<20	<20	<20	<20	<20	<20	**40**
	Mar 7	AI13-538	AHY	BWTE	<20	<20	<20	<20	<20	<20	<20	<20	<20	**20**	<20	**160**	**160**
	Mar 7	AI13-541	AHY	BWTE	<20	**80**	**80**	**40**	<20	<20	**640**	**160**	<20	**80**	**160**	160	**20**
	Mar 7	AI13-554	AHY	BWTE	<20	<20	<20	<20	<20	<20	<20	<20	<20	<20	<20	<20	**20**
	Mar 15	AI13-1002	AHY	BWTE	<20	<20	<20	**20**	<20	<20	**40**	<20	<20	**20**	**40**	<20	**80**
	Mar 15	AI13-1072	AHY	AGWT	<20	<20	<20	<20	**20**	<20	<20	<20	<20	<20	<20	<20	**20**
	Aug 28	AI13-3025	AHY	MALL	<20	**20**	<20	<20	<20	<20	<20	**20**	<20	<20	**40**	<20	**20**
	Aug 28	AI13-3035	AHY	MALL	<20	<20	<20	<20	<20	<20	<20	<20	<20	<20	<20	**40**	**20**
	Aug 28	AI13-3109	HY	MALL	<20	**20**	**80**	<20	<20	<20	<20	<20	<20	<20	<20	<20	**80**
	Sep 4	AI13-3115	AHY	MALL	<20	<20	<20	<20	<20	<20	<20	<20	<20	<20	<20	<20	**80**
	Sep 4	AI13-3123	AHY	MALL	<20	<20	<20	<20	<20	<20	<20	<20	<20	<20	<20	**40**	**20**
	Sep 4	AI13-3125	HY	MALL	<20	<20	<20	<20	<20	<20	<20	<20	<20	<20	<20	<20	**20**
	Sep 12	AI13-3209	HY	MALL	<20	<20	<20	<20	<20	<20	<20	<20	<20	<20	<20	**40**	**20**
2014																	
	Mar 7	AI14-49	AHY	BWTE	**20**	**80**	<20	<20	<20	<20	**640**	**20**	<20	**160**	**40**	**20**	**20**

*The volume of sera for 2 of the H14 positive samples was inadequate for testing against the complete panel of antigens. HA subtypes are organized based on phylogenetic relationships because patterns of cross-reactivity would be expected on the basis of clade membership. Case-patients are identified by sample ID.
†Abbreviations for age are: UA, unknown age; HY, hatched-year bird; AHY, after hatched-year bird.
‡Abbreviations for species are: MALL, mallard; BWT, blue-winged teal; AGWT, American green-winged teal.
§Virus neutralization antibody titers of <20 indicate negative results and titers of >20 are considered positive. Bold numbers indicate values >20.
